# Testing and Validation of the Vehicle Front Camera Verification Method Using External Stimulation

**DOI:** 10.3390/s24248166

**Published:** 2024-12-21

**Authors:** Robin Langer, Maximilian Bauder, Ghanshyam Tukarambhai Moghariya, Michael Clemens Georg Eckert, Tibor Kubjatko, Hans-Georg Schweiger

**Affiliations:** 1 CARISSMA Institute of Electric, Connected, and Secure Mobility (C-ECOS), Technische Hochschule Ingolstadt, Esplanade 10, 85049 Ingolstadt, Germany; maximilian.bauder@thi.de (M.B.); hans-georg.schweiger@thi.de (H.-G.S.); 2 Faculty of Electrical Engineering and Information Technology, Technische Hochschule Ingolstadt, Esplanade 10, 85049 Ingolstadt, Germany; 3 Institute of Forensic Research and Education, University of Zilina, 010 26 Zilina, Slovakia; kubjatko@uniza.sk

**Keywords:** ADAS, front camera, high beam assist, periodic technical inspection (PTI), simulation, Tesla Model 3, test drives, testing, Volkswagen ID.3, Volkswagen T-Cross

## Abstract

The perception of the vehicle’s environment is crucial for automated vehicles. Therefore, environmental sensors’ reliability and correct functioning are becoming increasingly important. Current vehicle inspections and self-diagnostics must be adapted to ensure the correct functioning of environmental sensors throughout the vehicle’s lifetime. There are several promising approaches for developing new test methods for vehicle environmental sensors, one of which has already been developed in our previous work. A method for testing vehicle front cameras was developed. In this work, the method is improved and applied again. Various test vehicles, including the Tesla Model 3, Volkswagen ID.3, and Volkswagen T-Cross, are stimulated by simulating driving scenarios. The stimulation is carried out via a tablet positioned before the camera. The high beam assist is used to evaluate the vehicle’s reaction. It was observed whether the vehicle switched from high to low beam as expected in response to the stimulation. Although no general statement can be made, the principle of stimulation works. A vehicle reaction can be successfully induced using this method. In further test series, the influence of display brightness is examined for the first time in this work. The results show that the display brightness significantly influences the test procedure. In addition, the method is validated by stimulation with colored images. It is shown that no complex traffic simulation is necessary to trigger a vehicle reaction. In the following validation approach, the CAN data of the Tesla Model 3 is analyzed during the tests. Here, too, the assumption that the vehicle reaction is based solely on the detected brightness instead of identifying road users is confirmed. The final validation approach examines the method’s applicability to other vehicles and high beam assist technologies. Although the method could not be used on the Volkswagen T-Cross due to a fault detected by the vehicle’s self-diagnosis, it worked well on the Volkswagen ID.3. This vehicle has a dynamic light assist in which individual segments of the high beam are dimmed during stimulation. Although the method developed to stimulate vehicle front cameras is promising, the specific factors that trigger the vehicle responses remain to be seen. This uncertainty suggests that further research is needed better to understand the interaction of stimulation and sensor detection.

## 1. Introduction

The periodic technical inspection (PTI) is a regular assessment of vehicles regarding traffic safety and environmental compliance. The PTI follows Directive 2014/45/EU [1] in the EU and is governed by the German Road Traffic Registration Ordinance (StVZO) and associated directives. The FSD Fahrzeugsystemdaten GmbH (FSD) [2] acts as the Central Agency for PTI commissioned by the Federal Republic of Germany, providing information and testing instructions. Only authorized inspectors [3] are permitted to conduct a PTI, during which the vehicle is examined thoroughly and non-destructively according to StVZO criteria. The results of the PTI depend on the identified malfunctions and their severity. If a vehicle has no malfunctions, it passes the PTI and receives a certificate and inspection sticker. Vehicles used for passenger transport must undergo re-inspection every two years [3].

With the increasing number of automated vehicles [4,5], the scope of PTI should also adapt [6,7,8,9,10]. A significant challenge is ensuring the correct functioning of environmental sensors essential for automated driving. This is crucial for safe traffic operations. Since 7 July 2024, EU Regulation 2019/2144 [11] has made installing ADAS and the necessary environmental sensors mandatory for first registrations.

The PTI, therefore, faces significant challenges [12]. New test methods are needed to test the environment sensors and the associated ADAS functions. The reliable functionality of these systems is essential, as drivers are often unable to react appropriately in the event of technical malfunctions [13].

In addition to the PTI, vehicle self-diagnosis is continuously carried out as a technical check by the vehicle itself by the manufacturer. However, related work has shown that vehicle self-diagnosis does not always work without errors [14,15].

There are already promising approaches to meet the need for new test methods for vehicle environment sensors and ADAS. In work in [16], camera detection, including the lighting system, is tested. The tests take place in a custom-built hall that allows reproducible variation of the light intensity, from complete darkness to full brightness. In addition, pairs of headlights can be adjusted in their horizontal distance to the test vehicle. [16]

The work by [17] presents methods for testing camera systems as a function of the development process. However, there is no consideration during vehicle operation. The options mentioned include simulations, hardware-in-the-loop (HiL) test benches, video interface boxes (VIB), and vehicle-in-the-loop (ViL) test benches [17].

FSD, together with DEKRA and other partners, has developed new test methods for automated driving functions and ADAS in the ErVast project [14]. The functionality of vehicle systems and environment sensors was tested in the project. A target-based approach was developed in which a dynamic target stimulates the environment sensors of a vehicle under test (VUT). It was determined whether the stimulation was correct. A framework developed in the project made it possible to connect the components and to control and evaluate test sequences. Overall, the project contributes to improving road safety through “Vision Zero”, a future without road fatalities [14].

The KÜS DRIVE (Dynamic Roadworthiness Inspection for VEhicles) [18,19] is a modern test bench for developing new test procedures for the PTI of automated vehicles. It includes various components such as radar target simulators and camera monitors to test the functionality and safety of the vehicles. A unique feature is the scenario-based efficiency test, which checks the reaction of the technical system to known stimuli without physical contact, supported by over-the-air technology. Vehicle-in-the-loop (VIL) simulation enables testing in a virtual environment. ADAS functions can also be tested. Despite its high cost and complexity, the test bench offers valuable opportunities for research and development of new test methods [19].

In our previous work [6], several concepts for testing a vehicle’s front camera were developed. One concept was selected as a cost-effective test method and implemented in a proof of concept. The results show that the test vehicle, a Tesla Model 3, reacts to simulation scenarios projected onto a screen in front of the vehicle, stimulating the vehicle’s front camera. With the high beam assistant activated, a complete vehicle reaction was detected by automatically switching off the high beam.

This work aims to improve the developed method and to demonstrate a proof of concept with new test series. Furthermore, the test method is to be validated with additional tests. The following research questions should, therefore, be answered with this work:

- RQ1: Is it possible to stimulate a sensor for vehicle environment perception with a virtual driving environment to trigger a complete vehicle reaction?
- RQ2: How realistic must the stimulation of the sensor for vehicle environment perception be to trigger a complete vehicle reaction?

## 2. Methods and Materials

### 2.1. Theoretical Background

Adaptive headlight systems are systems for driving light control. These include dynamic bend lighting, static bend lighting (cornering light), automatic headlight control, and the high beam assist. These systems improve the driver’s vision, significantly increasing safety at night [20,21,22].

Night driving accounts for 60% less traffic, but 40% of all serious accidents [23]. Additionally, the accident rate is higher at night compared to daytime, and these accidents tend to be more severe [24]. This is influenced by the fact that around 90% of the information drivers receive is visual [25], and their visual perception is significantly worse at night. A well-illuminated environment is crucial for the driver while ensuring that other road users are not subjected to glare. According to the Technical Inspection Association (TÜV SÜD) [26], low beam headlights are one of the most common defects found during periodic technical inspections [27]. Since some headlight systems depend on additional sensors, such as the high beam assist relying on the front camera, it is crucial that they are regularly inspected to ensure their functionality.

As an ADAS system, high beam assist automatically adapts the high beam to the driving environment by deactivating and reactivating the high beam. The vehicle reaction depends on various conditions. The high beam assist deactivates the high beam under the following conditions [21]:

- Other road users in the proximity;
- Strong ambient lighting (e.g., urban areas);
- The vehicle speed falls below a threshold value $v_{deactivate}$.

Accordingly, high beam assist is only active when no other road user has been detected and the ambient lighting is sufficiently dark. It is also necessary for the vehicle to exceed a speed threshold $v_{activate}$ to activate the high beam assist. The vehicle can collect and process the necessary data with integrated environmental (exteroceptive) sensors. The vehicle uses the data to decide whether the conditions are met to activate or deactivate the high beam assist. The conditions are often not visible to independent third parties. Partial information can occasionally be found in the respective vehicle manuals [28,29,30].

An advanced version of the high beam assist is the so-called glare-free high beam, also known as dynamic light assist [31]. This system dynamically adjusts segments of the high beam light cone. When a road user is detected, the high beam is only deactivated in the relevant segments to prevent glare. The rest of the surroundings remain illuminated. AUDI AG, for example, refers to this system as Matrix LED Headlight [32].

### 2.2. Experimental Setup and Design

**Figure 1:**
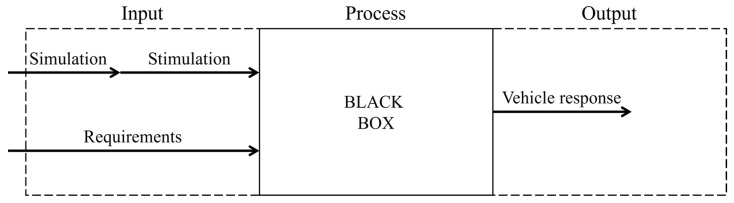
The method is based on the input-process-output principle. Simulations are used to stimulate a vehicle. Specific requirements are necessary for this. The vehicle and its interpretation are considered as a black box. The vehicle reaction is observed via the high beam assist [6].

In this work, our method for testing a vehicle front camera from our previous work [6] is to be enhanced, applied, and validated. The method, shown in Figure 1, is based on the input-process-output principle.

**Figure 2:**
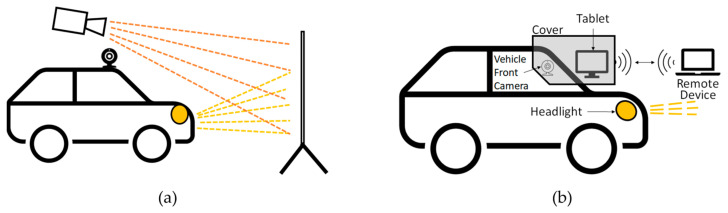
Conceptual representation of the two setups: (**a**) shows the setup of the previous work, in which the simulation was projected onto a screen via a beamer [6]. (**b**) In this work, a tablet is mounted in front of the VUT camera and covered with a cardboard box to block ambient light. The tablet can then be controlled by a remote device.

In our previous work [6], the vehicle front camera was stimulated by a simulation projected onto a screen or bridge pillar wall in front of the vehicle via a projector. A conceptual sketch can be seen in Figure 2a. The vehicle was seen as a black box, as independent third parties usually have no intrinsic information about the vehicle. The vehicle reaction is to be determined using the high beam assist. Specific requirements were necessary for this, such as the already introduced speed threshold values $v_{activate}$ and $v_{deactivate}$. The evaluation is based on a pass or fail result. If the vehicle with activated high beam assist automatically deactivates the high beam during stimulation, it successfully passes the test. If the high beam is not automatically deactivated so that there is no vehicle reaction, it has failed the test. Manually switching on the high beam is not part of this work. The following is always about the high beam assist, which must be activated and can then automatically activate or deactivate the high beam.

Our previous work [6] has shown that the test setup can be affected by ambient light. With the white screen initially used, the reflection of the vehicle’s high beam was so intense that the vehicle perceived too much ambient light and deactivated the high beam without any simulation. Although using the bridge pillar wall improved the setup, ambient light could still not be completely ruled out.

**Figure 3:**
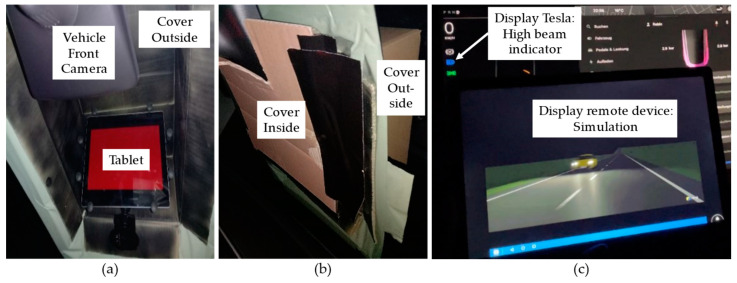
Setup of the VUT. (**a**) View from inside the vehicle, showing the tablet positioned in front of the front camera. (**b**) Similar viewpoint as (**a**), but with a cover made of cardboard boxes. (**c**) View of the Tesla monitor, highlighting the high beam indicator lamp and the overlapping remote device displaying a simulation scenario.

In the enhanced approach of this work, a tablet is mounted on the windshield to stimulate the vehicle’s front camera so that the vehicle’s front camera can detect the tablet screen. A conceptual sketch can be seen in Figure 2b, and the actual setup in Figure 3. A RAM Mounts X-Grip tablet holder is used for mounting [33]. The tablet is secured in the holder and adjusted along the windshield until the center of the tablet screen is as closely aligned as possible with the midpoint of the vehicle’s front camera. The positioning was performed by eye. The tablet and its holder are covered with a cardboard box outside the windshield. The cardboard is fixed to the outside of the windshield with duct tape. The tape is roughly positioned, with one part adhering to the cardboard itself and the other securing it to the windshield. Inside the vehicle, part of the windshield is also covered with cardboard and taped with duct tape to close possible gaps, see Figure 3b. This minimizes the intrusion of ambient light from inside and outside light sources. In addition, the cardboard boxes used are painted black inside to minimize light reflections from the tablet itself. Furthermore, the rain and light sensors, usually located close to the vehicle’s front camera, were also covered with tape to ensure that only the vehicle’s front camera was stimulated. Overall, this makes it possible to isolate the front camera and the tablet and eliminate as many interfering factors as possible. The tablet in front of the front camera is operated via a wireless remote device connected to the tablet.

As in our previous work [6], the vehicle is a black box, and recording its reaction via the high beam assists in using the pass/fail result, which remains identical. The costs are comparable to the original method, with both being low-cost. Some components from the original testing method are no longer needed, such as the projector, the laptop connected to it, and the generator for the mobile operation of the projector. In the new method, only a tablet with a holder, a cardboard cover, and another device to remote control the tablet is required, easing the new method’s handling. The setup is also simpler because, in the original method, the position in front of the wall screen had to be approached with the vehicle every time after the high beam was activated, and the projector with the laptop had to be positioned on the stationary vehicle. In contrast, the tablet is already mounted on the vehicle during the activation drive, and the camera can be stimulated immediately once the high beam is activated. The new method is more reliable because the shielding significantly reduces the amount of ambient light, which would otherwise lead to unintended stimulation of the camera.

#### 2.2.1. Test Series 1: Simulation Scenarios

**Figure 4:**
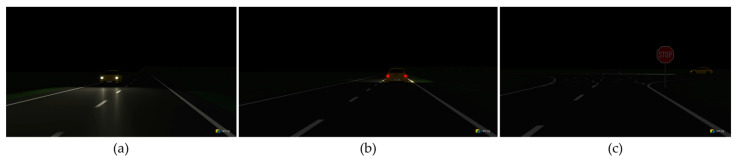
Example frames of the three simulation scenarios used, created in [6]. (**a**) passing an oncoming vehicle that has its low beam activated. (**b**) a vehicle driving ahead with activated taillights. (**c**) entry of an intersecting vehicle in which both a headlight and a taillight are visible.

Simulation scenarios already created in [6] were used again in this work. These virtual driving scenarios were created with CarMaker (Version 10.0) [34]. The three different simulation scenarios comprehensively cover driving scenarios likely to occur in real driving situations. Figure 4 shows one frame of each of the three simulation scenarios. The ego vehicle in the simulation scenario, which represents the VUT, is realized from a point of view (POV) so that the body of the ego vehicle is not visible. In this case, the applicability of the simulation scenarios is increased due to the independence from the VUT. As the tests are to be carried out in the dark and the vehicle headlights could have a significant influence, the simulations are also carried out in the dark.

In the first simulation scenario (a), an oncoming vehicle in the opposite lane passes the POV on the left. The passing vehicle has an activated low beam. Approximately 19 s after passing, another identical vehicle passes by. This has the advantage that the simulation can be continued if the VUT does not deactivate the high beam at the first vehicle. The subsequent passing vehicle can be used as another test sample. This can significantly reduce the test time, especially for vehicles with adaptive high beam assist. In the second simulation scenario (b), the POV of the VUT approaches a leading vehicle in front with red taillights. In the third simulation scenario (c), a crossing vehicle from the right has activated a headlight and a taillight. The POV of the VUT waits at the STOP sign until the crossing vehicle has left the intersection. These three simulation scenarios were chosen to perform the stimulation using vehicle headlights, rear lights, and a combination.

A Tesla Model 3 (2021) was used as the VUT. The speed threshold value $v_{activate}=50\;km/h$ was determined in prior driving tests. The speed threshold value $v_{deactivate}$ does not exist for the Tesla, as the high beam assist with activated high beam remains activated even when stationary.

The test was therefore carried out as follows: The VUT was modified with the respective tablet and the cover. The tablet was placed in the center in front of the front camera by eye. The VUT was accelerated to $v_{activate}=50\;km/h$ so that the high beam assist was activated, and the high beam was activated automatically. This was followed by moderate braking to a standstill. A simulation scenario was then played via the remote device on the tablet in front of the vehicle’s front camera, thus stimulating the camera. The vehicle’s reaction could be determined immediately using the light pattern of the headlights and the high beam indicator lamp, see Figure 3c.

The tablet, housed within the cover, was remotely controlled via a wireless connection from a mobile phone. This was achieved by installing the TeamViewer Quick Support application on the tablet, becoming a remotely controllable device. The companion app, TeamViewer Remote Control, was installed on the mobile phone, allowing it to serve as the controlling device. Additionally, the mobile phone functioned as an internet-providing hotspot to which the tablet was connected. Note that the remotely controlled device (the tablet) must be an Android device, as iOS devices are not supported for remote control by the TeamViewer application [35]. However, Android and iOS devices can control the Android tablet, permitting adjustments to internal settings such as screen brightness.

The tests were carried out on a rural, non-illuminated road [36] at night. During the test, no other road users were in proximity, and no light cones for other road users were visible. A Samsung Tab A (2016) tablet was used for the first series of tests. Each simulation scenario was run three or more times for high reproducibility, and a vehicle reaction was documented.

#### 2.2.2. Test Series 2: Simulation Scenarios with Different Brightness

The influence of tablet brightness on vehicle response was investigated for the second test series. For this purpose, the same set-up was used but with the two other tablets, Samsung A8 Tablet and Google Pixel C Tablet, and an adapted test procedure. With the two different tablets, an attempt was also made to compensate for the camera’s detection sensitivities and the technological limitations of the tablet displays. The brightness of the tablets was varied during the tests. Five levels with equidistant intervals were defined from minimum to maximum brightness. Level 1 corresponds to a brightness of 0%, level 2 approx. 25%, level 3 approx. 50%, etc. A new test sample always started at brightness level 1, where a simulation scenario was run and a vehicle reaction documented. If there was no reaction from the VUT (high beam remained activated), the tablet’s brightness was increased to the next level, and the same simulation was rerun. This was repeated until a vehicle reaction was observed or there was no reaction at maximum tablet brightness. Again, each simulation scenario was run three times per tablet.

#### 2.2.3. Validation 1: Color Images with Different Brightness

**Figure 5:**
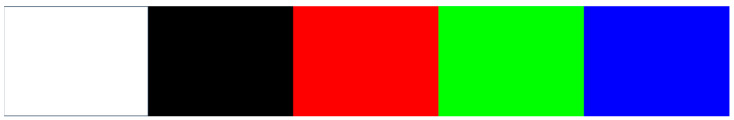
The chosen color images, white, black, red, green, and blue, to stimulate the vehicle’s front camera.

To validate the test method, the aim was to determine how realistic the stimulation of the vehicle front camera must be to trigger a vehicle reaction (automatic deactivation of the high beam). The setup and test procedure of this validation 1 are identical to test series 2, except that color images are now displayed on the tablet instead of the simulation scenarios. For this purpose, the three additive primary colors, red (1, 0, 0), green (0, 1, 0), and blue (0, 0, 1), and additionally black (0, 0, 0) and white (1, 1, 1), were selected from the RGB color spaces. The selected color pictures can be seen in Figure 5.

These five color images were created with the Microsoft Paint software (version 11.2304.33.0) and displayed on the respective tablet as borderless as possible. After activating the high beam assist and activating the high beam automatically, the tablet was set to minimum brightness as usual. If no vehicle reaction could be detected, the tablet’s brightness would slowly and continuously increase via the remote device. At the same time, the static color image remained the same until the high beam was automatically deactivated. The brightness level was documented accordingly. The tests were carried out three times for each of the five color images.

Additionally, the brightness of the color images of the respective tablets was measured with a lux meter “MS200” [37]. For this purpose, the respective color image was displayed on the tablet with the set brightness level in a dark environment. The lux meter was aligned with the tablet at approximately 10 cm. The five color images with each brightness level were measured three times on two tablets, and the values were arithmetically averaged.

#### 2.2.4. Validation 2: CAN Bus Data

To further validate the test method, signals from the CAN (controller area network) bus of the Tesla Model 3 were recorded and evaluated. The CAN bus of the Tesla Model 3 was evaluated only for successful automatic deactivation of the high beam as a vehicle reaction to the stimulation. The recorded data could be interpreted with a DBC file (DataBase Controller) [38] for the Tesla Model 3. The CAN data was recorded and evaluated three times for each simulation scenario, with the high beam automatically deactivated.

Two signals with the following possible states were evaluated in the CAN data feed of the Tesla Model 3:

- DAS High Low Beam Decision
  - 0 “DAS HIGH BEAM UNDECIDED”
  - 1 “DAS HIGH BEAM OFF”
  - 2 “DAS HIGH BEAM ON”
  - 3 “DAS HIGH BEAM SNA”
- DAS High Low Beam Off Reason
  - 0 “HIGH BEAM ON”
  - 1 “HIGH BEAM OFF REASON MOVING VISION TARGET”
  - 2 “HIGH BEAM OFF REASON MOVING RADAR TARGET”
  - 3 “HIGH BEAM OFF REASON AMBIENT LIGHT”
  - 4 “HIGH BEAM OFF REASON HEADLIGHT”
  - 5 “HIGH BEAM OFF REASON SNA”

The “Decision” signal can track when an automatic change from high to low beam occurs. The “Reason” signal can then trace the reason for the change.

#### 2.2.5. Validation 3: Applicability to Other Vehicles

This final validation 3 determines whether the test method can be applied to other vehicles and high beam assist technologies. For this purpose, tests with a VW T-Cross and a VW ID.3 were supplemented. The high beam assist was initially operated in real traffic in darkness for all three test vehicles. No irregularities were observed.

For the VW T-Cross, the three simulation scenarios were also used to stimulate the front camera. The test setup was identical to the one used for the Tesla. For the T-Cross, the speed thresholds were determined with $v_{activate}=35\;km/h$ and $v_{deactivate}=20\;km/h$. As with the Tesla, the tests on the T-Cross were carried out at night on a rural road [36] without other road users or lighting.

**Figure 6:**
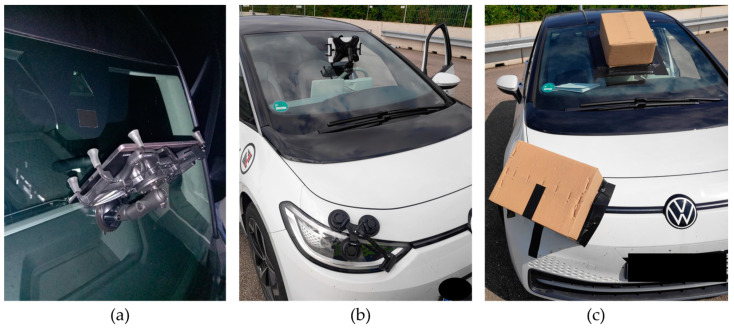
Test vehicle setups: (**a**) the positioning of the tablet and its mount in front of the VW T-Cross’ front camera. Below the front camera, the tape covering the light/rain sensor is visible. (**b**) the positioning of the tablet and its mount in front of the VW ID.3’s front camera. Additionally, a GoPro camera was attached to the headlight; (**c**) the VW ID.3 with the tablet and GoPro camera covered.

A speed threshold value $v_{deactivate}=20\;km/h$ was determined for the VW ID.3. Therefore, it is not possible to perform the tests at a standstill with this VUT. The tests were carried out at the CARISSMA Outdoor Test Facility [39], which is only possible during the day. Despite being carried out during the day, the masking of the light/rain sensor and the cover was sufficient for the high beam assist to be activated. The speed threshold value $v_{activate}=60\;km/h$ was exceeded on the acceleration section of the test facility, and the high beam was thus automatically activated. After activation, the vehicle drove in a constant circle at approx. 22 km/h so that the high beam assist and the high beam remained activated. Unlike the Tesla Model 3, the VW ID.3 has a different high beam assist technology, the dynamic light assist, so only individual segments of the high beam are deactivated. Accordingly, the indicator lamp does not go out, so the vehicle reaction must be determined via the headlight pattern. Adjustments to the test setup were necessary to detect the headlight pattern during the day. The previous setup in front of the vehicle’s front camera remained the same as in the previous tests. However, a GoPro HERO8 Black was attached to the headlight, see Figure 6b. The GoPro camera faces away from the headlight. As can be seen in Figure 6c, the GoPro and the spotlight are also covered with cardboard. The Go-Pro camera is filming the light pattern cast by the headlight on the inside of the cardboard box. The Samsung Tab A (2016) tablet was always set to maximum brightness. The three simulation scenarios were used for stimulation. Each simulation scenarios were tested multiple times.

## 3. Results

### 3.1. Test Series 1: Simulation Scenarios

First, the tests with the Tesla Model 3 showed that the automatically activated high beam remains activated even when braking to a standstill. If the parking position is activated, the high beam is deactivated, but the high beam assist immediately activates it when switching back to driving mode.

Table 1 shows the results of stimulating the front camera of the Tesla Model 3 with the three simulation scenarios. Automatic deactivation of the high beam counts as a detected vehicle reaction. No vehicle reaction could be observed if the high beam remained activated during the entire stimulation.

In the simulation with an oncoming vehicle, the Tesla only reacted to the stimulation once and automatically deactivated the high beam. Six further stimulations failed to trigger a vehicle reaction. In contrast, the high beam was successfully deactivated automatically eleven out of eleven times when simulating a leading vehicle. In the last simulation of a crossing vehicle, the Tesla showed no vehicle reaction out of three trials.

### 3.2. Test Series 2: Simulation Scenarios with Different Brightness

Table 2 and Table 3 show the results of the test series 2. In Table 3, with the Google Pixel C tablet, the vehicle tends to deactivate the high beam at a lower brightness level than the Samsung A8 tablet in Table 2. However, in all cases, there was a vehicle reaction before the maximum brightness of the tablets was reached. The oncoming vehicle simulation is the same for both tests. In the simulation with the leading vehicle, the VUT reacts at a higher brightness level when stimulated with the Samsung A8 tablet. In contrast, the Google Pixel C tablet reacts at even lower brightness. A similar behavior can be seen in the third simulation scenario with the vehicle from the side.

### 3.3. Validation 1: Color Images with Different Brightness

Table 4 and Table 5 provide an overview of the color images with the corresponding brightness levels to which the VUT reacted. The results are almost identical for both tables. The high beam deactivation always occurs at level 2, with only one exception for white, red, and green. However, the blue display leads to a high beam deactivation for both tables only at level 3. On the other hand, black is the only color image that stands out from the other color images. A vehicle reaction could only be triggered once out of 3 trials with both tablets. There was no vehicle reaction in 2 tests with the black color image on each tablet, even at maximum brightness. Table 6 and Table 7 show the measured light intensity values of the lux meter for the respective tablet.

### 3.4. Validation 2: CAN Bus Data

The data recorded from the Tesla Model 3 CAN bus is in hex format. Using the DBC (Database Container) file [38], the individual signals could be interpreted. During the analysis, the interpreted signals “DAS High Low Beam Decision” and “DAS High Low Beam Off Reason” were exported and analyzed for each test run. The time series of the signal “DAS High Low Beam Decision” was first examined to determine the time of the signal change from “HIGH BEAM ON” to “HIGH BEAM OFF.” At this time stamp, the other signal, “DAS High Low Beam Off Reason”, could be used to determine the reason for the deactivation of the high beam assist.

The evaluation of the recorded CAN bus data with automatic deactivation of the high beam shows “DAS HIGH BEAM OFF” for the “DAS High Low Beam Decision” signal in all trials without exception. This confirms that the VUT deactivated the high beam during the tests, as observed visually by the headlight pattern and indicated by the control light. Likewise, without exception, the value “HIGH BEAM OFF REASON AMBIENT LIGHT” resulted in the signal “DAS High Low Beam Off Reason” for all test samples.

### 3.5. Validation 3: Applicability to Other Vehicles

No results could be obtained for the VW T-Cross. After the setup with the tablet and the cover was completed, the ignition was started. Immediately after starting the ignition of the vehicle, information was displayed in the driver’s cockpit that the windshield should be cleaned (original text in German: “Light Assist: Bitte Frontscheibe reinigen”.). In this case, the vehicle diagnostics of the VUT recognized something in front of the vehicle’s front camera. The ignition of the VUT was switched off and on again several times, but the error message was displayed each time. Therefore, the tests with the VW T-Cross had to be aborted at this point.

**Figure 7:**
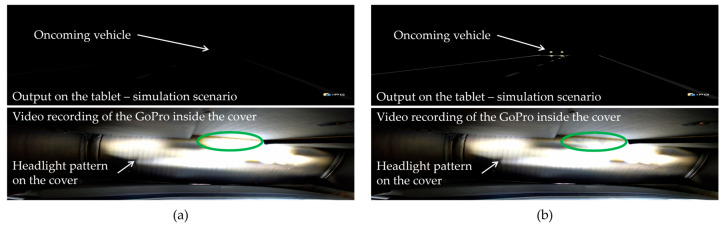
Results from application on additional vehicles: (**a**,**b**) **Top**: Output of the simulation scenario on the tablet; (**a**,**b**) **bottom**: GoPro camera recording of the dynamic light beam pattern from inside the cardboard box cover. The dimmed segment is visible in the area marked in green.

In contrast, the VW ID.3 produced obvious results, as shown in Table 8. In the simulation of an oncoming vehicle, the VUT deactivated the high beam in a particular segment in 28 of the 28 trials carried out for this scenario. For both the simulation of the leading vehicle and the simulation of the vehicle from the side, 16 trials were carried out, none of which produced a vehicle reaction. Figure 7a,b shows the output of the simulation scenario on the tablet in the upper part. The respective simultaneous camera feed of the GoPro camera facing the headlight pattern can be seen in the partial image below. A headlight pattern can be seen on the inner surface of the cardboard cover. Significant changes occurred in the area marked in green as the oncoming vehicle drove past our POV in the simulation. The headlight beam in the area marked in green was significantly dimmed as the simulated vehicle drove past and then illuminated again after the vehicle had passed. A difference can be seen when comparing the green area from partial images (a) and (b) in Figure 7. While the vehicle is further away in (a), the area marked in green is fully illuminated by the high beam. In (b), the simulated vehicle is probably detected, and the high beam is then dimmed in the area marked in green. This could be reproduced for all 28 trials.

## 4. Discussion

The results of test series 1 on the Tesla Model 3 can be compared directly with the results of the proof of concept on the Tesla Model 3 from [6] and with validation 3 on the VW ID.3. The simulation scenarios were played at constant brightness in all three test series. In [6] the stimulation was carried out via a projector in test series 1 of this work via a tablet on the Tesla Model 3 and in validation 3 via a tablet on the VW ID.3. The stimulation with the projector worked reproducibly in all three simulation scenarios. With one exception, vehicle reactions could always be triggered. Here, the type of light source in the simulation (headlights or taillights) does not appear to influence the result. In contrast, when stimulated via a tablet on the Tesla Model 3, only the simulation scenario with the leading vehicle could always trigger a vehicle reaction without exception. With the oncoming vehicle and vehicle from the side, no vehicle reaction could be triggered, with one exception. Here, it appears that Tesla is particularly sensitive to the red taillights of a leading vehicle when stimulated by a tablet. Surprisingly, there is no vehicle reaction for the vehicle from the side with only one taillight. This could be because the size of the individual taillight always remains the same during the simulation. In contrast, the size of the taillights on the leading vehicle increases as the VUT approaches the leading vehicle. With the VW ID.3, on the other hand, a different behavior can be observed. Apparent results were obtained despite a high number of test trials. The larger headlights of the oncoming vehicle are presumably particularly effective here, as a vehicle reaction could always be triggered.

RQ1 can be answered with the discussed results: Yes, stimulating a sensor for vehicle environment perception with a virtual driving environment is sufficient to trigger a complete vehicle reaction. Depending on the VUT used, the simulation scenarios can be more or less effective in triggering a vehicle reaction.

In both test series 2 and validation 1 with the color images, the results between the Samsung A8 tablet and the Google Pixel C tablet barely differ. There is only a slight trend that the Google Pixel C Tablet can trigger a vehicle reaction even at lower brightness levels. This is consistent with the light intensity measurements taken in validation 1. The light intensity of the Google Pixel C Tablet tends to be slightly higher compared to the Samsung A8 Tablet for all colors. In particular, the light intensity at the first brightness level of the white color image is more than twice as high, and at the fifth brightness level of the white color, the image is almost twice as high as on the Samsung A8 tablet.

In test series 1, two scenarios—an oncoming vehicle and a vehicle from the side—did not trigger a vehicle reaction. The Samsung Tab A (2016) tablet was used in these tests. In the tests from test series 2, other tablets were used, such as the Samsung A8 tablet and the Google Pixel C tablet. Here, a vehicle reaction could be triggered with each simulation scenario. Not even the maximum brightness of the tablet was necessary for this. The Samsung Tab A (2016) may have a lower brightness than the other two tablets. Unfortunately, the light intensity was not measured for the Samsung Tab A (2016), so it can only be speculated about.

The RQ2 can be answered with validation 1: The stimulation of a sensor for vehicle environment perception does not have to be realistic to trigger a complete vehicle reaction, at least in the case of the vehicle’s front camera. Simple color images are sufficient to trigger the reaction. Traffic scenarios for the simulation are not required to trigger a vehicle reaction using this method.

It is also interesting to note that the VW T-Cross recognized the test setup as a fault immediately after the ignition was switched on and the high beam assistant was reasonably deactivated. In the VW ID.3, another model from the same manufacturer, and in the Tesla Model 3, the self-diagnosis shows no error. Accordingly, no assistance functions are restricted, which is questionable given the camera’s limited field of view due to the test setup.

However, even after the tests, it remains unclear whether the VUT deactivates the high beam due to object recognition, i.e., actually recognizes the simulated vehicle as such or only reacts due to brightness. The necessity of utilizing the tablet’s operating system overlay to adjust the display brightness of the tablet in front of the front camera has already led to changes in brightness. During the tests, it occasionally happened that the high beam was already deactivated when the operating system overlay was opened. At least with the Tesla Model 3, the can data confirms the assumption that the vehicle reaction is based solely on a change in the ambient brightness. The CAN data shows that in all samples from validation 2, the vehicle reaction is due to the ambient light. A CAN message was expected with “HIGH BEAM OFF REASON MOVING VISION TARGET” or “HIGH BEAM OFF REASON HEADLIGHT”.

The Tesla Model 3 also allows the image from the front camera to be shown on the proprietary display in the vehicle interior. After the test setup, this view shows that the tablets were visibly placed in front of the front camera, but the display is blurred. The front camera will most likely not be designed to place the focal point at such a close distance, making it even more questionable whether the vehicle reaction is based on a recognized vehicle. Moreover, manually positioning the tablet by eye is a method that is difficult to reproduce accurately. While Tesla’s proprietary display with the front camera image allows for almost borderless positioning, this feature is not available in every vehicle and is time-consuming.

As an independent third party with no intrinsic knowledge of the vehicle, data processing in the vehicle can only be regarded as a black box. This is seen as the biggest problem for the test method used. For reliable tests, the tester should know what the vehicle perceives and interprets via the perception of the environment. Notably, the Tesla Model 3 is equipped with several additional cameras (such as those on the side mirrors, B-pillars, and rear) that are not stimulated using this method. Since the vehicle is treated as a black box, how perceived environmental data is processed remains unknown. Sensor data fusion seems possible, where the front camera detection (simulation scenario) might contradict the detections from other environmental sensors (real stationary environment). This discrepancy could lead to potential error messages, depending on how the data is processed. This issue already arises with stereo cameras, as the method does not account for such technologies. Stereo cameras that detect the tablet screen cannot provide depth information, leading to possible issues in data processing and vehicle response. Consequently, this method cannot determine whether the high beam deactivation timing is correct, too early, or too late. Also, this method cannot verify whether the dynamic high beam assist is dimming the correct segment. If an additional sensor is unintentionally stimulated, for instance, by an interfering vehicle or a person at the test site, the test must be repeated as a precaution. This is necessary because the detection of additional traffic participants is very likely to be captured by other sensors, thus directly influencing the data processing. This precautionary measure is time-consuming. An interface that reads a live sensor data feed from all vehicle environment sensors would suit this purpose. In addition, a read-out intention or calculated behavior of the vehicle based on the recorded data would be desirable. An elaboration of this interface and the definition of its scope is helpful here.

Furthermore, the method’s validation is limited to the three vehicles used in this work. The method could be successfully validated for the Tesla Model 3 and VW ID.3; this was impossible with the VW T-Cross. Although the three test vehicles used are sufficient for this proof of concept, further research must show the method’s suitability for other vehicles and whether generally valid statements can be derived from it.

Currently, this method is unsuitable for PTI, as it cannot fully determine the vehicle reaction’s basis. In addition, the time required, especially with actual test drives, is too long for the PTI framework. An interface for activating assistance systems at a standstill without having to fulfill the usual conditions would simplify testing significantly.

However, it should always be considered that testing on its own does not guarantee error-free performance [40]. The primary goal should be to make automated vehicles safer and to ensure the functionality of environmental detection. To this end, alternative approaches should be pursued in addition to the test method described in this work. These include, for example, improving the vehicle’s own diagnostics or concretely developing standardized test methods.

## 5. Conclusions

In this work, we continued the test method we developed in an earlier work [6] for testing a vehicle’s front camera. The aim was to stimulate the front camera of a complete vehicle by using simulation. A vehicle reaction was detected with the high beam assist. The test was passed if the high beam was automatically deactivated during the simulation.

As part of this work, the original test method was enhanced—the amount of ambient light and interfering factors could be reduced to a minimum. This was achieved by covering the new test setup. The stimulation is performed using a tablet in front of the front camera instead of a projector with a screen.

Two test series were carried out. A vehicle reaction could be triggered by automatically deactivating the high beam by stimulating the front camera using simulation scenarios. From this, RQ1 can be answered: The stimulation of a sensor for vehicle environment perception with a virtual driving environment is sufficient to trigger a complete vehicle reaction.

Three independent approaches were chosen to validate this test method. The first validation showed that abstract stimulation, for example, by a color image, is already sufficient to trigger a vehicle reaction. RQ2 can, therefore, be answered: The stimulation of a sensor for vehicle environment perception does not have to be realistic to trigger a complete vehicle reaction.

The second validation analyzed the CAN bus signals of the Tesla Model 3 regarding the high beam assist. As a result, it was observed that the automatic deactivation of the high beam is due to ambient light. Possible other signals, such as moving target or headlight, were not recorded. This leads to the assumption that the vehicle reaction is due to a change in brightness and not based on actual recognized objects or road users.

In a third validation phase, the method was applied to the Volkswagen ID.3. This resulted in changes in the vehicle type and the high beam assist technology. The ID.3 comes with a dynamic light assist. During the tests using the test method, a change in the high beam cone segment was identified. Overall, the applicability of a Volkswagen ID.3 was successful. However, an application in a Volkswagen T-Cross could not be examined, as the vehicle’s self-diagnosis gave an error due to the test setup.

In general, the testing method has several limitations, which make it impractical to integrate into the current state of periodic technical inspections (PTI). The most significant problem with the method is that the data processing in the vehicle must be treated as a black box for independent third parties. It is often impossible to understand what environmental data the vehicle collects and how it is processed. It, therefore, remains mostly unclear why the vehicle reacted in our test trials. Although a reproducible reaction could be triggered with the test method, it remains theoretically conceivable that further unintentional stimulation, for example, of the other environmental sensors, could have triggered a vehicle reaction. Besides, the method is also too time-consuming.

In summary, new test methods are required for independent functional testing of vehicle environment sensors and ADAS functions to ensure their functionality throughout vehicle operation. Vehicle manufacturers will eventually have to provide more information about the vehicles so that appropriate test methods can be used.

## Figures and Tables

**Table 1:** Results of the stimulation with simulation scenarios with the Tesla Model 3. Vehicle reaction observed means that the Tesla automatically deactivated the high beam. No vehicle reaction observed means the Tesla high beam remained activated during the test.

Tesla Model 3	Experimental Trials—Simulation Scenarios		
Samsung Tab A (2016)	Vehicle Reaction Observed	No Vehicle Reaction Observed	Total
Oncoming Vehicle	1	6	7
Leading Vehicle	11	0	11
Vehicle from side	0	3	3

**Table 2:** Results of stimulating the front camera of the Tesla Model 3 with the three simulation scenarios. “o” means no vehicle reaction could be observed. “x” means a vehicle reaction could be observed by changing from high beam to low beam. Samsung A8 Tablet was used as a tablet.

Tesla Model 3	Experimental Trials—Simulation Scenarios with Different Brightness								
Samsung A8 Tablet	Oncoming Vehicle			Leading Vehicle			Vehicle from side		
Tablet Brightness Level	# 1	# 2	# 3	# 1	# 2	# 3	# 1	# 2	# 3
1	o	o	o	o	o	o	o	o	o
2	o	o	o	o	o	o	x	o	o
3	x	x	x	o	x	o		x	x
4				x		x			
5									

**Table 3:** Results of stimulating the front camera of the Tesla Model 3 with the three simulation scenarios. “o” means no vehicle reaction could be observed. “x” means a vehicle reaction could be observed by changing from high beam to low beam. Google Pixel C Tablet was used as a tablet.

Tesla Model 3	Experimental Trials—Simulation Scenarios with Different Brightness								
Google Pixel C Tablet	Oncoming Vehicle			Leading Vehicle			Vehicle from side		
Tablet Brightness Level	# 1	# 2	# 3	# 1	# 2	# 3	# 1	# 2	# 3
1	o	o	o	o	o	o	o	o	o
2	o	o	o	x	o	o	x	x	x
3	x	x	x		x	x			
4									
5									

**Table 4:** Experimental trials with color images with increasing brightness of the Samsung A8 tablet. “o” means no vehicle reaction could be observed. “x” means a vehicle reaction could be observed by changing from high beam to low beam.

Tesla Model 3	Experimental Trials—Color Images with Different Brightness														
Samsung A8 Tablet	White			Black			Red			Green			Blue		
Tablet Brightness Level	# 1	# 2	# 3	# 1	# 2	# 3	# 1	# 2	# 3	# 1	# 2	# 3	# 1	# 2	# 3
1	o	o	o	o	o	o	o	o	o	o	o	o	o	o	o
2	x	x	x	o	o	x	x	x	x	x	x	x	o	o	o
3				o	o								x	x	x
4				o	o										
5				o	o										

**Table 5:** Experimental trials with color images with increasing brightness of the Google Pixel C tablet. “o” means no vehicle reaction could be observed. “x” means a vehicle reaction could be observed by changing from high beam to low beam.

Tesla Model 3	Experimental Trials—Color Images with Different Brightness														
Google Pixel C Tablet	White			Black			Red			Green			Blue		
Tablet Brightness Level	# 1	# 2	# 3	# 1	# 2	# 3	# 1	# 2	# 3	# 1	# 2	# 3	# 1	# 2	# 3
1	o	x	o	o	o	o	o	o	o	o	o	o	o	o	o
2	x		x	x	o	o	x	x	x	x	x	x	o	o	o
3					o	o							x	x	x
4					o	o									
5					o	o									

**Table 6:** Measurement of light intensities using a lux meter on color images captured with a Samsung A8 Tablet.

Lux Meter	Experimental Trials—Color Images with Different Brightness				
Samsung A8 Tablet	White	Black	Red	Green	Blue
Tablet Brightness Level	Avg. in Lux	Avg. in Lux	Avg. in Lux	Avg. in Lux	Avg. in Lux
1	5.05	1.59	1.36	4.29	2.86
2	70.53	1.50	11.67	50.10	9.58
3	118.60	1.56	18.85	90.27	13.85
4	163.77	0.88	24.08	123.70	18.92
5	220.90	1.29	33.06	167.73	25.62

**Table 7:** Measurement of light intensities using a lux meter on color images captured with a Google Pixel C Tablet.

Lux Meter	Experimental Trials—Color Images with Different Brightness				
Google Pixel C Tablet	White	Black	Red	Green	Blue
Tablet Brightness Level	Avg. in Lux	Avg. in Lux	Avg. in Lux	Avg. in Lux	Avg. in Lux
1	12.93	0.13	2.02	9.35	1.61
2	11.60	0.31	13.99	66.23	8.34
3	172.97	1.02	23.60	118.53	14.15
4	243.03	0.51	32.97	168.20	19.31
5	403.67	1.29	52.70	277.50	32.22

**Table 8:** Results of the stimulation with simulation scenarios with the Volkswagen ID.3. Vehicle reaction observed means that the ID.3 automatically dimmed a segment in the dynamic light pattern. No vehicle reaction observed means that the ID.3 high beam remained static during the test.

Volkswagen ID.3	Experimental Trials—Simulation Scenarios		
Samsung Tab A (2016)	Vehicle Reaction Observed	No Vehicle Reaction Observed	Total
Oncoming Vehicle	28	0	28
Leading Vehicle	0	16	16
Vehicle from side	0	16	16

## Data Availability

The data presented in this study are available on request from the corresponding author.
